# ﻿A new species of *Ceratostema* (Ericaceae) with nearly tubular leaves from the Cordillera del Cóndor, Ecuador

**DOI:** 10.3897/phytokeys.261.156555

**Published:** 2025-08-07

**Authors:** Marco M. Jiménez, Henry X. Garzón-Suárez, Gabriel A. Iturralde

**Affiliations:** 1 Grupo de Investigación en Biodiversidad, Medio Ambiente y Salud BIOMAS, Carrera de Ingeniería Agroindustrial, Facultad de Ingeniería y Ciencias Aplicadas, Universidad de Las Américas, UDLA, Vía a Nayón, Quito 170124, Ecuador Universidad de Las Américas Quito Ecuador; 2 Grupo Científico Calaway Dodson: Investigación y Conservación de Orquídeas del Ecuador, Quito, 170510, Pichincha, Ecuador Grupo Científico Calaway Dodson: Investigación y Conservación de Orquídeas del Ecuador Pichincha Ecuador; 3 Herbario HUTPL, Departamento de Ciencias Biológicas, Universidad Técnica Particular de Loja, San Cayetano Alto s/n 11-01-608, Loja, Ecuador Universidad Técnica Particular de Loja Loja Ecuador; 4 Jungle Dave’s Science Foundation, San Juan Bosco, Ecuador Jungle Dave’s Science Foundation San Juan Bosco Ecuador

**Keywords:** Northern Andes, Morona Santiago, south-eastern Ecuador, taxonomy

## Abstract

A new species of *Ceratostema* from the Cordillera del Cóndor in Ecuador is described and illustrated. *Ceratostemarevoluta* is distinguished by its nearly tubular leaves with revolute margins, pulvinate petioles, reddish flowers with white base and brownish to black lobes, short, complanate pedicel with carinate bracteoles appressed to the base and calyx lobes appressed to the corolla and overlapping each other below the middle. The taxonomic similarities of the new species are discussed and information about its distribution, habitat and conservation is provided.

## ﻿Introduction

Ericaceae are a large and diverse family of flowering plants comprising ca. 125 genera and over 4,250 species, distributed predominantly in temperate and montane tropical regions (Luteyn 2002; Christenhusz and Byng 2016). About 800 species are present in the Neotropics, most of which belong to tribe Vaccinieae, comprising 30 genera (Luteyn 2002). In Ecuador, 17 of these genera are present, including the Andean genus *Ceratostema* Juss. (Luteyn 2021). *Ceratostema* includes 40 species with 36 accepted species in Ecuador (Jiménez et al. 2024d; POWO 2025). Nine new species have been recently described (Cornejo and Luteyn 2024; Doucette et al. 2024, 2025a, b, c, d); with these new additions, the number of species of the genus currently increases to 45 in this country.

Members of *Ceratostema* are distinguished from the other genera of Vaccinieae by the presence of an articulation between the pedicel and hypanthium, flowers with large corollas and relatively elongate lobes. Other additional distinguishing characters present in *Ceratostema* are the stamens equal in length to the corolla, with coarsely papillate thecae and the anther tubules about half the diameter of the thecae (Luteyn 2005). The species of this genus are epiphytic or terrestrial shrubs distributed in Ecuador from 450 to 3950 m in elevation, inhabiting lowland and montane forests, inter-Andean valleys up to paramo areas (Luteyn 1996).

The Cordillera del Cóndor is a mountain range located on the border between Ecuador and Peru, extending 150 km from north to south and reaching the highest elevation at ca. 2900 m (Neill 2005). From exploration and taxonomic work, new taxa have been described in recent years (Clark and Neill 2023; Jaramillo 2023; Jiménez et al. 2024b, c; Molino et al. 2025). The latest explorations in the region have led to the discovery of new species of Ericaceae, such as *Ceratostemaingridportillae* A. Doucette, H. Medina & J. Portilla, *Disterigmachriscanadayi* Cornejo & Luteyn and *Sphyrospermumgrandiflorum* Cornejo & Pedraza (Cornejo and Pedraza 2019; Cornejo et al. 2024; Doucette et al. 2025b). During fieldwork conducted since 2022, MJ and HG found an additional species of *Ceratostema* southeast of Morona Santiago Province, which, after further investigation, was found to be new to science and is here illustrated and described.

## ﻿Materials and methods

The original descriptions of similar species (Luteyn 1996; Doucette et al. 2024; Jiménez et al. 2024a) were reviewed and compared to the characters of the new species. The scanned image of the original material of *Ceratostemabracteolatum* Luteyn was obtained through the Bioweb database (https://bioweb.bio/portal/) stored at the Catholic University Herbarium (**QCA**).

Fresh flowers and leaves were preserved in 70% ethanol, 29% water and 1% glycerol. Measurements of the vegetative and floral parts were made from the living plants and preserved material. Digital images were taken with a Nikon D3100 camera with an AF-S VR Micro-Nikkor 105mm f/2.8G IF-ED lens. The type and examined specimens of the new species were collected under permit No. MAATEDB I-CM-2022-0248, granted by the Ministerio del Ambiente y Transición Ecológica del Ecuador (**MAATE**). The geographic coordinates of the specimens were omitted for conservation purposes; detailed data can be consulted in the herbarium voucher.

## ﻿Taxonomic treatment

### ﻿ Ceratostema revoluta

Taxon classificationPlantaeEricalesEricaceae

M.M.Jiménez, H.Garzón & Iturralde sp. nov.

92698485-F256-50E9-A897-66DFE6A0CA62

urn:lsid:ipni.org:names:77366806-1

Figs 1 , 3

#### Diagnosis.

**Figure 1.**
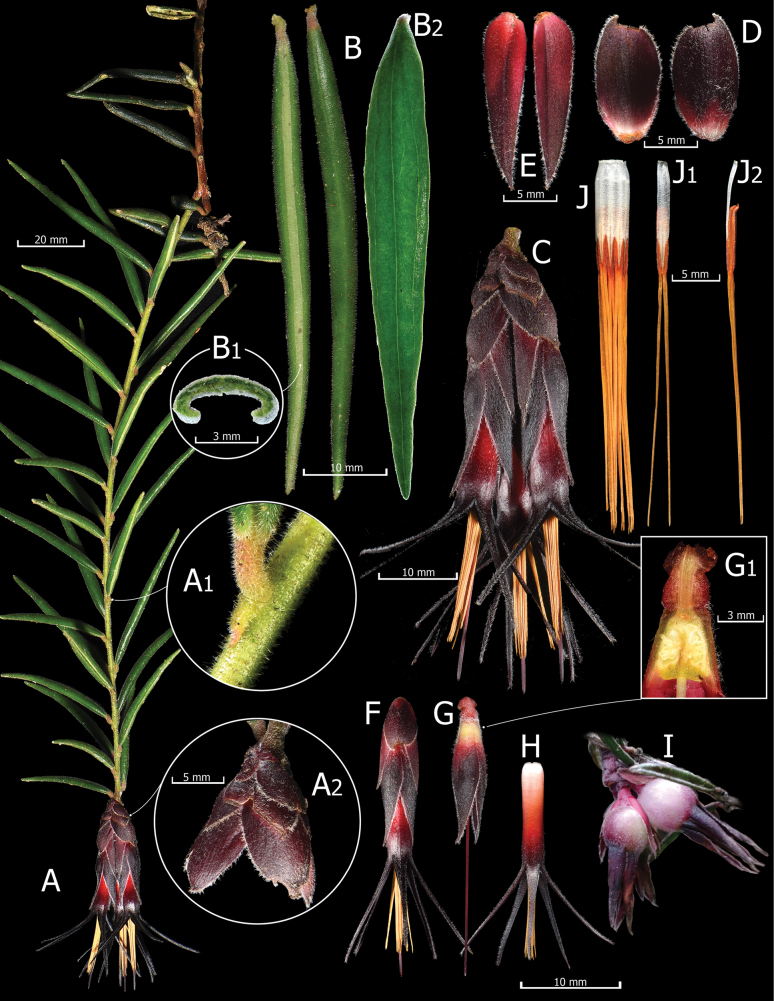
*Ceratostemarevoluta*. **A.** Fertile branch, with petiole of leaf with axillary bud (**B**) and rachis bracts (**C**); **D.** Abaxial (left) and adaxial (right) views of leaves with transverse section of leaf (**E**) and expanded leaf showing venation under backlighting (**F**); **G.** Inflorescence; **H.** Bracts, abaxial (left) and adaxial (right) views; **I.** Bracteoles, adaxial (left) and abaxial (right) views; **J.** Flower; **K.** Hypanthium, calyx, pedicel and style, with longitudinal section of pedicel, hypanthium, nectariferous disc and ovary (**L**); **M.** Corolla; **N.** Mature fruits; **O.** Stamens, dorsal (**P**) and lateral (**Q**) views. Prepared by G.A. Iturralde and H.X. Garzón-Suárez from photographs of the holotype.

*Ceratostemarevoluta* is distinguished from other members of the genus by its nearly tubular, linear-lanceolate leaves with revolute margins, pulvinate petioles, sessile inflorescences with reddish flowers with white base and brownish to black lobes, short complanate pedicels with basal carinate appressed bracteoles and appressed calyx lobes that overlap each other below the middle (Fig. 1).

#### Type.

Ecuador • Morona-Santiago: Cerca de San Juan Bosco, 1298 m elev., 8 Mar 2024, *H. Garzón 265* (**holotype**: HUTPL!).

#### Description.

Erect, epiphytic ***shrubs***; indumentum of subpersistent trichomes, arranged unevenly, white, eglandular, 0.3–0.9 mm long, sparse to dense on younger branches, petioles, leaf blades, inflorescences and flowers, excluding stamens and style; roots axonomorphous, with well-developed lignotubers, lignotubers subspherical to fusiform, 5.6–8.5 × 4.3–7.4 cm, 13.4–17.3 cm in diameter. ***Stems*** terete to subterete, slightly arching, up to 1 m long, glabrous, arising from the lignotuber; older stems dark brown, cracking longitudinally and exfoliating; younger branches pendent, slightly arcuate, pale green, terete, ca. 25.7 cm long, 2.8 mm wide, puberulous, becoming striate and dark brown when old or after exfoliation; axillary buds 2–3, emerging 1 mm below leaf node, foliar bracts pale pink, narrowly triangular, 0.9–1.2 × 0.7 mm, puberulous. ***Leaves*** spirally arranged, suberect to nearly horizontal; petioles pale pink, pulvinate, 2.1–3.0 × 1.3–1.7 mm, puberulous; blades dark green and somewhat lustrous adaxially, paler abaxially, linear-lanceolate, nearly tubular, 2.0–5.7 × 0.4 cm, thinly coriaceous, puberulent adaxially and tomentulose abaxially, glabrescent, mid-vein impressed adaxially, conspicuous and raised abaxially, venation obscure, 3-plinerved from near base and reticulate against a backlight, base cuneate, margins strongly revolute and longitudinally curled, apex acute. ***Inflorescence*** supraxillary, sessile, congested, 4–8 mm × 4.1–5.9 mm, 3–9-flowered; rachis obconic, rugose, covered by bracts, tomentose; bracts persistent, up to 10, dark brownish-red, paler towards base, ovate to transversely ovate, 3.0–14.2 × 3.6–9.5 mm, apex obtuse; bracts persistent, similar in colour and texture to bracts, ovate, 16.5–21.0 × 6.3–10.5 mm, acute to attenuate; pedicel dark red, complanate, 4.0–4.4 × 3.1–3.6 mm, tomentose; articulation present between pedicel and hypanthium; bracteoles persistent, 2, located near middle, oppositely arranged, dark brownish-red, long ovate-triangular, 15.2–17.6 × 3.9–4.3 mm, channelled abaxially, centrally carinate adaxially, margins long-ciliate, apex attenuate. ***Flowers*** pentamerous, pendulous; hypanthium green, except margins pale purple, reddish towards apex, obconic, obscurely pentagonal, 3.0–4.2 × 4.0–5.7 mm, tomentose; calyx erect, open, 12.7–21.5 × 4.0–7.0 mm, tomentose, hairs white, limb 0.4–1.3 × 4.0–5.7 mm; lobes 5, nearly reaching throat of corolla, overlapping in basal half, black, except reddish towards base, lanceolate, slightly convex, 12.3–20.2 × 4.0–5.4 mm, tomentose, base attenuate, margin long-ciliate, apex acuminate, sinuses acute. ***Corolla*** white-tomentose on apical half, whitish at base, reddish-brown in middle, tubular, except slightly dilated proximally and expanded distally, 4.0–5.1 cm long (including lobes), 4.5 mm in diameter at base and throat, thick-carnose, bistratose; lobes 5, spreading, black, narrowly linear-triangular, 15.9–22.5 × 2.3–2.8 mm, lustrous, tomentose externally, glabrous internally, channelled and subverrucose internally, slightly recurved to base, apex acute. ***Stamens*** 10, nearly equalling corolla, in two series somewhat unequal in length, 3.7–4.7 cm long; filaments connate, each separated by a longitudinal line dorsally, white,10.4–12.7 mm long, glabrous and lustrous on both sides; anthers 3.2–4.3 cm long, thecae conspicuously papillose, 6.6–7.7 mm long, prognathous with a basal appendage ca. 0.4 mm long; tubules distinct, but apparently connate in proximal 5/6, 2.5–3.2 cm long, glabrous, dehiscing by terminal pores, ca. 1.1 × 0.2 mm. ***Style*** exserted, longer than stamens, dark reddish-brown, 4.1–4.7 cm long, glabrous, base white; stigma truncate. ***Fruit*** a berry, purplish when ripe, globose, ca. 12.8 × 12.9 mm in diameter, pubescent, calyx lobes persistent.

#### Distribution and habitat.

**Figure 2.**
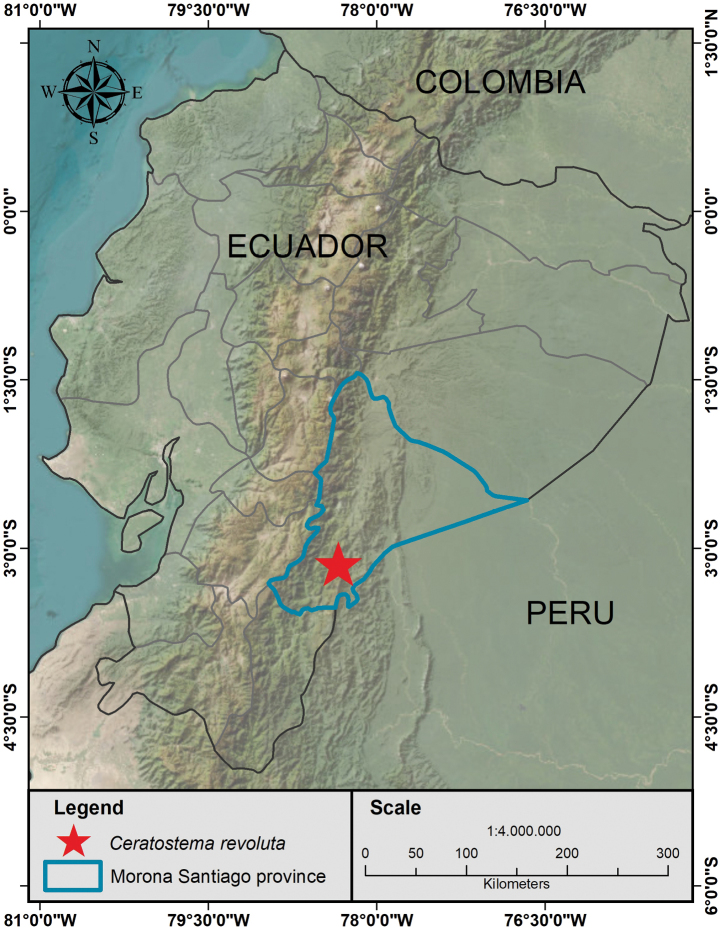
Geographic distribution of *Ceratostemarevoluta*. Prepared by H.X. Garzón-Suárez.

*Ceratostemarevoluta* has been reported in the south-eastern Province of Morona Santiago (Fig. 2). The species is only known from the steep western foothills of the northern part of the Cordillera del Condor, where it was found in the region east of San Juan Bosco at elevations between 1300 and 1700 m.

#### Etymology.

The new species is named by the strongly revolute margins of the leaves.

#### Taxonomic discussion.

**Figure 3.**
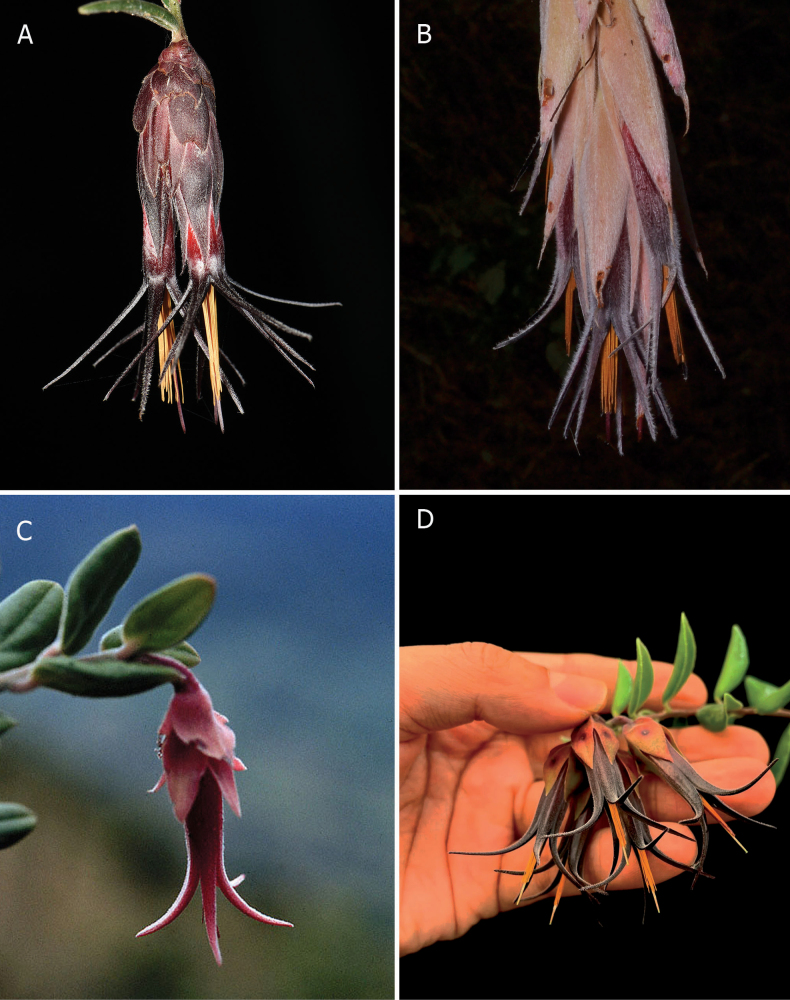
Species of *Ceratostema* with similar flowers. **A.***Ceratostemarevoluta*; **B.***C.agettiorum*; **C.***C.bracteolatum*; **D.***C.doucettei*. **A, B.** Photographed by H.X. Garzón-Suárez; **C.** By G. Harling; **D.** By A. Ito.

*Ceratostemarevoluta* is similar to *C.agettiorum* M.M. Jiménez & H. Garzón, *C.bracteolatum* and *C.doucettei* H. Medina & J. Portilla in being variously covered with indumentum, the mostly sessile inflorescence, the conspicuous calyx lobes with acute sinuses, the darkly coloured corollas (Fig. 3) and the stamens with the filaments connate, but can be distinguished by various characters (Table 1). From this group, the new species is most similar to *C.agettiorum* in the conspicuous floral bracts covering the rachis of the inflorescence, the pentamerous flowers with the calyx erect, the acuminate calyx lobes and the nearly tubular corollas with the lobes black, straight half-spreading. The new species is distinguished from *C.agettiorum* by the erect stems (vs. pendent), the smaller, 2.0–5.7 × 0.4 cm, linear-lanceolate, nearly tubular leaf blades (vs. 7.1–15.3 × 4.0–8.4 cm, broadly ovate, flat), leaf blades cuneate at the base (vs. cordate) and acute at the apex (vs. attenuate), leaf blades obscurely 3-plinerved at the base (vs. strongly 7–11-plinerved), the sessile inflorescence with up to 9 flowers per raceme (vs. short-pedunculate, up to 15 flowers), the floral bracts 16.5–21.0 × 6.3–10.5 mm, ovate, acute, dark brownish-red (vs. 46.9–49.4 × 20.3–21.5 mm, elliptic, caudate, pink), the pedicel 4.0–4.4 mm long, torulose, red (vs. 10 mm long, obconic, pink), the bracteoles 15.2–17.6 × 3.9–4.3 mm (vs. 41.3–41.6 × 7.6–7.8 mm), carinate adaxially, attenuate (vs. convex, long acuminate), the hypanthium obscurely pentagonal (vs. 10-ribbed), the calyx black (vs. magenta), the corolla expanding distally (vs. narrowing distally), pubescent in the apical half including the lobes (vs. tomentose), the corolla white towards the base (vs. reddish-brown) and the filaments white, forming a tube (vs. pink, dilated towards the base) (Jiménez et al. 2024a).

**Table 1.** Morphological comparison of *Ceratostemarevoluta* and similar species. References taken from: (1) presented herein, (2) Jiménez et al. (2024); (3) Luteyn (1996) and (4) Doucette et al. (2024).

Characters	*C.revoluta* (1)	*C.agettiorum* (2)	*C.bracteolatum* (3)	*C.doucettei* (4)
Habit	Erect, epiphytic	Pendant, epiphytic	Climbing, epiphytic or terrestrial (?)	Erect to ascending, epiphytic
Indumentum on vegetative and floral parts	Puberulous on vegetative parts, tomentose on floral parts	Puberulous on vegetative parts and tomentose on floral parts	Short-pilose	Pubescent
Petioles	2.1–3.0 mm long, pulvinate, pale pink	6.3–8.4 mm long, terete, pale green	3.2–3.9 mm long, subterete, pale red	3.0–4.0 mm long, subterete, pale green
Leaves	Linear-lanceolate, 2.0–5.7 × 0.4 cm, semi-tubular, obscurely 3-plinerved, apex acute, base cuneate, margins strongly revolute and longitudinally curled	Broadly-ovate, 7.1–15.3 × 4.0–8.4 cm, flat, strongly 7–11-plinerved, apex attenuate, base cordate, margins slightly revolute	Elliptic-ovate, 3–6 × 1.5–2.5 cm, convex, 5–7-plinerved, apex attenuate, base rounded or obtuse, margins revolute and longitudinally curled	Ovate to narrowly ovate, 3.7–4.9 × 1.2–2.0 cm, flat to convex, pinnately veined, apex subacute to obtuse, base obtuse, margins revolute
Inflorescence	3–9-flowered, sessile	Up to 15 flowers, short-pedunculate	Solitary or rarely 2–4-flowered, sessile	Few-flowered, sessile
Flower merosity	Pentamerous	Pentamerous	Pentamerous	Tetramerous
Floral bracts	16.5–21.0 × 6.3–10.5 mm, ovate	46.9–49.4 × 20.3–21.5 mm, elliptic	4.0–6.0 × 3.0–4.0 mm, ovate	ca. 1.8–2.0 mm long, triangular
Pedicel	4.0–4.4 mm long, complanate, red	10 mm long, obconic, pink	13–19 mm long, coarsely angled to subterete, red	6.5–7.2 mm long, terete, purple
Bracteoles	15.2–17.6 × 3.9–4.3 mm long, ovate-triangular, attenuate, median	41.3–41.6 × 7.6–7.8 mm, narrowly ovate, acuminate, median	13.0–20.0 × 12.0–18.0 mm, broadly ovate, short acuminate, apical	Size unknown, scale-like, basal
Hypanthium	3.0–4.2 × 4.0–5.7 mm, obconic, obscurely pentagonal	5.3–6.1 × 3.6–5.2 mm, obconic, 10-ribbed	7.0–10.0 × 7.2 mm, obprismatic, weakly, but distinctly 10-costate	5.0–6.2 × 5.0–6.9 mm, obconic, 5-winged
Calyx lobes	Lanceolate, apex acuminate, 12.3–20.2 × 4.0–5.4 mm, sinuses acute	Narrowly lanceolate, apex acuminate, 17.8–19.2 × 3.7–3.9 mm, sinuses acute	Ovate, apex acuminate, 12–17 × 7–10 mm, sinuses acute	Narrowly triangular, apex acute, 16.0–18.0 × 5.0–6.0 mm long, sinuses acute
Corolla	Reddish-brown and black, white towards base, tubular, but expanded distally, 4.0–5.1 cm long, pubescent on apical half	Dark scarlet or black, tubular, but slightly narrowing distally, 4.7–5.0 cm long, tomentose on apical half	Red, cylindrical, not ventricose, 4.2–4.4 cm long, glabrous where covered by calyx, but short-pilose distally	Reddish-purple, dark purple towards apex, tubular, weakly inflated towards base, pubescent, 2.3–2.6 cm long (excluding lobes)
Corolla lobes	Narrowly linear-triangular, acute,15.9–22.5 mm long, black, half-spreading	Linear-lanceolate, acute, 18.4–25.0 mm long, dark scarlet and black, half-spreading	Linear-lanceolate, ca. 19 mm long, purplish, reflexed	Narrowly triangular, long-acuminate, dark purple, spreading
Stamens	3.7–4.7 cm long; filaments connate into a tubular corona, white, 10.4–12.7 mm long; thecae 6.6–7.7 mm long	4.1–4.5 cm long, filaments connate into a dilated to the base corona, pink with white base, 9.9 mm long; thecae 5.0–6.0 mm long	3.7–3.8 cm long; filaments tubular connate into a tubular corona, ca. 6 mm long, thecae ca. 13.0–14.0 mm long	Ca. 7.0 cm long; filaments connate into a dilated to the base corona, whitish-green suffused with purple, 8.8–10.0 mm long; thecae 17.9–18.3 mm long
Style	Exserted, 4.1–4.7 cm long, dark reddish-brown	Not exserted, 4.7–4.9 cm long, pink, black above upper half	Not exserted, ca. 4.0 cm long	Exserted, ca. 4.3 cm long, whitish-green

#### Conservation status.

*Ceratostemarevoluta* is only known from several individuals in the southeast of Morona Santiago near San Juan Bosco. This region is strongly threatened by deforestation for cattle pastures, slash and burn agriculture, mining, and infrastructure works. We recommend *Ceratostemarevoluta* to be characterised provisionally as Data Deficient (DD) according to the IUCN Red List (IUCN 2024).

## Supplementary Material

XML Treatment for Ceratostema revoluta

## ﻿Additional information

### Use of AI

No AI-assisted technologies were used during the preparation of this manuscript.

### Author ORCIDs

Marco M. Jiménez https://orcid.org/0000-0002-9502-5651

Henry X. Garzón-Suárez https://orcid.org/0000-0003-4049-1652

Gabriel A. Iturralde https://orcid.org/0000-0003-2456-0929

### Data availability

All of the data that support the findings of this study are available in the main text.
